# Phase I randomized clinical trial of N-acetylcysteine in combination with an adjuvant probenecid for treatment of severe traumatic brain injury in children

**DOI:** 10.1371/journal.pone.0180280

**Published:** 2017-07-07

**Authors:** Robert S. B. Clark, Philip E. Empey, Hülya Bayır, Bedda L. Rosario, Samuel M. Poloyac, Patrick M. Kochanek, Thomas D. Nolin, Alicia K. Au, Christopher M. Horvat, Stephen R. Wisniewski, Michael J. Bell

**Affiliations:** 1 Department of Critical Care Medicine, University of Pittsburgh School of Medicine, Pittsburgh, Pennsylvania, United States of America; 2 Department of Pediatrics, University of Pittsburgh School of Medicine, Pittsburgh, Pennsylvania, United States of America; 3 Safar Center for Resuscitation Research, University of Pittsburgh School of Medicine, Pittsburgh, Pennsylvania, United States of America; 4 Children’s Hospital of Pittsburgh of UPMC, Pittsburgh, Pennsylvania, United States of America; 5 Clinical and Translational Science Institute, University of Pittsburgh, Pittsburgh, Pennsylvania, United States of America; 6 Department of Pharmacy and Therapeutics, Center for Clinical Pharmaceutical Sciences, School of Pharmacy, University of Pittsburgh, Pittsburgh, Pennsylvania, United States of America; 7 Department of Environmental and Occupational Health, University of Pittsburgh, Pittsburgh, Pennsylvania, United States of America; 8 Department of Epidemiology, University of Pittsburgh, Pittsburgh, Pennsylvania, United States of America; 9 Department of Neurological Surgery, University of Pittsburgh School of Medicine, Pittsburgh, Pennsylvania, United States of America; Public Library of Science, FRANCE

## Abstract

**Background:**

There are no therapies shown to improve outcome after severe traumatic brain injury (TBI) in humans, a leading cause of morbidity and mortality. We sought to verify brain exposure of the systemically administered antioxidant N-acetylcysteine (NAC) and the synergistic adjuvant probenecid, and identify adverse effects of this drug combination after severe TBI in children.

**Methods:**

IRB-approved, randomized, double-blind, placebo controlled Phase I study in children 2 to 18 years-of-age admitted to a Pediatric Intensive Care Unit after severe TBI (Glasgow Coma Scale [GCS] score ≤8) requiring an externalized ventricular drain for measurement of intracranial pressure (ICP). Patients were recruited from November 2011-August 2013. Fourteen patients (n = 7/group) were randomly assigned after obtaining informed consent to receive probenecid (25 mg/kg load, then 10 mg/kg/dose q6h×11 doses) and NAC (140 mg/kg load, then 70 mg/kg/dose q4h×17 doses), or placebos via naso/orogastric tube. Serum and CSF samples were drawn pre-bolus and 1–96 h after randomization and drug concentrations were measured via UPLC-MS/MS. Glasgow Outcome Scale (GOS) score was assessed at 3 months.

**Results:**

There were no adverse events attributable to drug treatment. One patient in the placebo group was withdrawn due to adverse effects. In the treatment group, NAC concentrations ranged from 16,977.3±2,212.3 to 16,786.1±3,285.3 in serum and from 269.3±113.0 to 467.9±262.7 ng/mL in CSF, at 24 to 72 h post-bolus, respectively; and probenecid concentrations ranged from 75.4.3±10.0 to 52.9±25.8 in serum and 5.4±1.0 to 4.6±2.1 μg/mL in CSF, at 24 to 72 h post-bolus, respectively (mean±SEM). Temperature, mean arterial pressure, ICP, use of ICP-directed therapies, surveillance serum brain injury biomarkers, and GOS at 3 months were not different between groups.

**Conclusions:**

Treatment resulted in detectable concentrations of NAC and probenecid in CSF and was not associated with undesirable effects after TBI in children.

**Trial registration:**

ClinicalTrials.gov NCT01322009

## Introduction

Traumatic brain injury (TBI) is a significant cause of morbidity and mortality worldwide and the primary cause of death in children in the U.S. [1]. To-date, no primary therapies for TBI have translated to practice, with a common denominator in failed clinical trials being a lack of site-specific therapeutic drug monitoring for proposed neuroprotective agents [2, 3]. Therapies directly targeting pathophysiologic consequences of TBI in order to improve neurological outcome are desperately needed.

N-acetylcysteine (NAC) is an antioxidant and putative neuroprotective agent [4, 5]. Biochemically, NAC serves as cysteine donor for the synthesis of the intracellular antioxidant glutathione (GSH) and thus has the capacity to increase the cysteine pool available for replenishment of GSH under conditions of oxidative stress, and can directly function as an antioxidant via its thiol group.[6] NAC is very hydrophilic (logD -5.4) [6, 7], and current reports suggest that NAC has limited capacity to passively cross the intact blood-brain barrier (BBB) [6]. NAC as monotherapy has established clinical utility for prevention of hepatotoxicity after acetaminophen/paracetamol overdose [8]. NAC has also been evaluated in clinical trials targeting neurological diseases, including autism [9], major depression [10], neonatal asphyxia [11], and neurodegenerative disease [12]. Importantly, NAC has shown promise in preventing sequelae from blast-induced mild TBI, presumably via its antioxidant properties in brain [13].

Probenecid has been used as an antimicrobial adjunct since World War II, increasing systemic exposure of antimicrobial agents via inhibition of drug elimination through membrane transporters including organic anion transporters 1 and 3 (OAT1 and 3) [14] found in both brain and kidney [15]. Probenecid can also prevent intracellular depletion of GSH via inhibition of ATP binding cassette membrane transporter C1 (ABCC1) mediated export of glutathionylated substrates on plasma membranes [16]. We discovered that NAC is a substrate for probenecid-inhibitable transporters, and that co-administration of probenecid increases NAC concentration in both brain and plasma via inhibition of OAT1 and 3 [14], thus offering additive mechanisms for therapeutic synergy.

Importantly both probenecid and NAC are in clinical use, are approved by the U.S. Food and Drug Administration (FDA), and have favorable safety profiles. Accordingly, we hypothesized that probenecid could be used as an adjuvant in combination with NAC to increase and/or maintain NAC concentration in brain after TBI. In this randomized, double-blind Phase I study we sought to determine whether NAC and probenecid administered systemically resulted in detectable drug levels in CSF, and whether the drugs used in combination resulted in attributable adverse events in children after severe TBI.

## Methods

### Study design and participants

We performed a randomized, double-blind, Phase I study of the combination of probenecid and NAC versus placebos in children 2 to 18 years-of-age after severe TBI (Glasgow Coma Scale [GCS] score ≤ 8) recruited from November 2011-September 2013 at a single, tertiary Children’s Hospital. All patients were admitted to the pediatric intensive care unit (PICU) on the pediatric neurocritical care/trauma service. The study was approved by the University of Pittsburgh Institutional Review Board and written informed consent was obtained from parents and/or legal guardians of all children enrolled in the study. In addition, a Data Safety Monitoring Board from the University of Pittsburgh evaluated the safety of the study throughout the time of enrollment. The Phase I trial was stopped based on duration of funding (R01 NS069247). The study was registered in ClinicalTrials.gov (Identifier NCT01322009).

The research team screened patients for eligibility for this study. Inclusion criteria included (i) age > 2 y and < 18 y, (ii) severe TBI (GCS ≤ 8) after resuscitation, (iii) placement of an EVD as a part of their standard care, and (iv) placement of a naso/orogastric feeding tube for medication administration. The trauma Attending Physician was consulted regarding the safety of administering medications into the stomach prior to approach for informed consent. Exclusion criteria included (i) examination consistent with brain death after resuscitation, (ii) documented pregnancy, (iii) contraindications to probenecid (including active status epilepticus, blood dyscrasias, administration of salicylates as standard care, documented urate stones, hypersensitivity to probenecid, age < 2 years), (iv) contraindications to NAC (known allergy to NAC), (v) lack of ability to obtain consent from both parents/guardians, and (vi) inability to start medications within 24 h from the time of injury.

### Randomization, masking, and drug administration

After obtaining written consent, children were randomized by the use of a blind envelope system to: (1) probenecid (initial: 25 mg/kg/dose; maintenance: 10 mg/kg/dose every 6 h for 11 doses; maximum dose 500 mg) and NAC (initial: 140 mg/kg/dose; maintenance: 70 mg/kg/dose every 4 h for 17 doses) or (2) placebo of equal volume and timing administered via naso/orogastric tube. The regimen for NAC was based on dosing for treatment of acetaminophen/paracetamol overdose, and the regimen for probenecid was based on dosing for use as an antibiotic adjuvant. Placebo contents included equal volumes and dosing regimens of lactose powder (for opacity) suspended in Ora-Plus and normal saline. Study drugs or placebo were prepared by a dedicated Research Pharmacist and administered by the bedside nurse. Clinicians caring for the child and all research staff–with the exception of the Research Pharmacist and the Data Manager at the Data Coordinating Center–remained blinded to study assignment. Research staff remained blinded until all data were entered into the research database and all analyses were performed on clinical samples. Compliance with the timed drug administration was monitored by the study team and deviations from expected drug delivery times > 1 hour were considered a protocol deviation.

### Clinical management protocol

All children were treated using a standardized neurocritical care protocol that has been previously described [17]. Briefly, all children with severe TBI received comprehensive care to rapidly stabilize and assess for injuries, mitigate secondary insults and promote optimal neurological outcome in accordance with published guidelines [18]. This protocol includes early endotracheal intubation, mechanical ventilation, and rapid correction/normalization of hemodynamic parameters. Once the children were stabilized, neurological assessment and imaging studies to determine the extent of injury were performed. All patients were maintained in a neutral position with their head midline and the head of the bed elevated 30° to improve cerebral blood return to the thorax. Placement of intracranial pressure (ICP) monitoring devices occurred as soon as was feasible and both intraparenchymal (IP) and externalized ventricular drain (EVD) monitors were placed within the same frontal lobe. EVD monitors were zeroed based at the tragus of the ear and continuously drained at 10 cm above the midbrain and IP monitors were zeroed at the level of the EVD, and used and maintained in accordance with the manufacturer’s instructions (Codman Neuro, New Brunswick, NJ). Other invasive monitoring devices (arterial catheters, central venous catheters) were placed as part of standard practice, and some children also underwent brain tissue oxygen monitoring using LICOX (Integra Neurosciences, NJ). The clinical protocol was developed to standardize practice for periods of ICP crises (generally defined as ICP ≥ 20 mmHg for ≥ 5 minutes). Step-wise escalations of care were implemented via a protocol, and included analgesia/sedation (fentanyl), neuromuscular blockade (vecuronium), mild hyperventilation (PaCO_2_ ~ 35 mm Hg), hyperosmolar therapies (mannitol or 3% NaCl) and barbiturate administration. As mentioned above, continuous CSF diversion was employed in all children.

### Sample and data collection

Samples (CSF and serum) were collected over 4 days and were obtained prior to initial drug administration, 1 hour after the first dose, and daily prior to subsequent dosing (trough levels). An additional CSF sample was obtained at 6 h after drug administration. CSF was immediately processed by centrifuging at 3000 ×*g* for 10 min and collecting supernatant. Blood was immediately centrifuged at 3000 ×*g* for 10 min and serum was obtained. All processed samples were frozen at -80°C for batch analysis.

Data including patient demographics, physiological parameters, and adverse events were recorded and entered into a database. Demographic information included age, patient sex, and various injury scores (Acute Injury Score, GCS) measured at the appropriate time period. Physiological parameters included hourly variables (temperature, mean arterial pressure [MAP], ICP, and cerebral perfusion pressure [CPP]) recorded within the medical record as well as results of other tests done as part of standard practice (arterial blood gases, electrolyte values and others) and gross assessment of therapies required for management of ICP [18]. A priori definitions of adverse events were developed (S1 Table) and the occurrence of these complications as well as discharge Glasgow Outcome Scale (GOS) score were determined by the Research Coordinator (RLB) and clinical investigator (MJB) prior to unblinding of the randomization scheme. Available serum was also used to quantify neuron specific enolase (NSE) and glial fibrillary acidic protein (GFAP), brain-specific biomarkers associated with acute brain injury in children, by ELISA [19, 20]. NSE levels were adjusted for hemolysis in serum samples as previously described [19].

### Quantification of NAC and probenecid in serum and CSF

Total NAC and probenecid concentrations were quantified in serum and CSF using ultra-high performance liquid chromatography-tandem mass spectrometry (UPLC-MS/MS) using validated assays [14]. NAC samples were prepared by adding deuterated internal standard (d3-NAC, Cambridge Isotopes, Andover, MA) to 50 μL plasma or CSF and samples were reduced by adding dithiothreitol, derivatized with n-ethylmaleimide, and proteins were precipitated with acetonitrile. The supernatant was then dried out under nitrogen and reconstituted with water before injecting onto the UPLC-MS/MS system. Probenecid samples were diluted with water, proteins were precipitated with acetonitrile, and ethacrynic acid (Sigma-Aldrich, St. Louis, MO) was added as an internal standard. NAC chromatographic separation was achieved using a porous graphitic carbon hypercarb column 3.0 μm (1.0 × 100 mm) and an acetonitrile-formic acid gradient on an Accela series UPLC system (Thermo, San Jose, CA). For probenecid, an acetic acid isocratic elution and a BEH C18 1.7 μm, 2.1×100 mm column was used on an Acquity UPLC system (Waters, Milford, MA). MS/MS detection of NAC-NEM (*m/z* 289.1→158.1) and probenecid (*m/z* 284.1→240.1*)* was performed on a TSQ Quantum Ultra mass spectrometer with a heated electrospray source (Thermo, San Jose, CA). NAC and probenecid assay linear ranges (r^2^ > 0.992) in serum and CSF were 10–10,000 ng/mL (NAC serum), 10–2,500 ng/mL (NAC CSF), 0.25–800 μg/mL (probenecid serum), and 0.25–100 μg/mL (probenecid CSF). Assays were validated in each matrix to ensure linearity, precision, and accuracy with acceptable intra-/inter-assay variability (<10.5%).

### Statistical analysis

Descriptive statistics were summarized as counts (percentages, %) for categorical data, mean ± standard deviation (SD) or median [interquartile range, IR] for normally or non-normally distributed continuous data as appropriate. Serum and CSF concentrations of NAC are presented as mean ± standard error of the mean (SEM). Fisher’s exact test was used to compare the frequency distribution of categorical variables between the groups. The Wilcoxon-Mann-Whitney test was performed to assess differences between groups for non-normally distributed continuous data. Linear mixed models were used to test the main effects of time (baseline, 1, 24, 48, 72, and 96 hours) and group (placebo and Pro-NAC), and time by group interactions on the brain injury biomarkers NSE and GFAP. All tests were two-sided and the significance level was defined at 0.05. Analyses were conducted using SAS, version 9.3 statistical software (SAS Institute Inc., Cary, NC; Operating System Windows 10).

## Results

**Fig 1:**
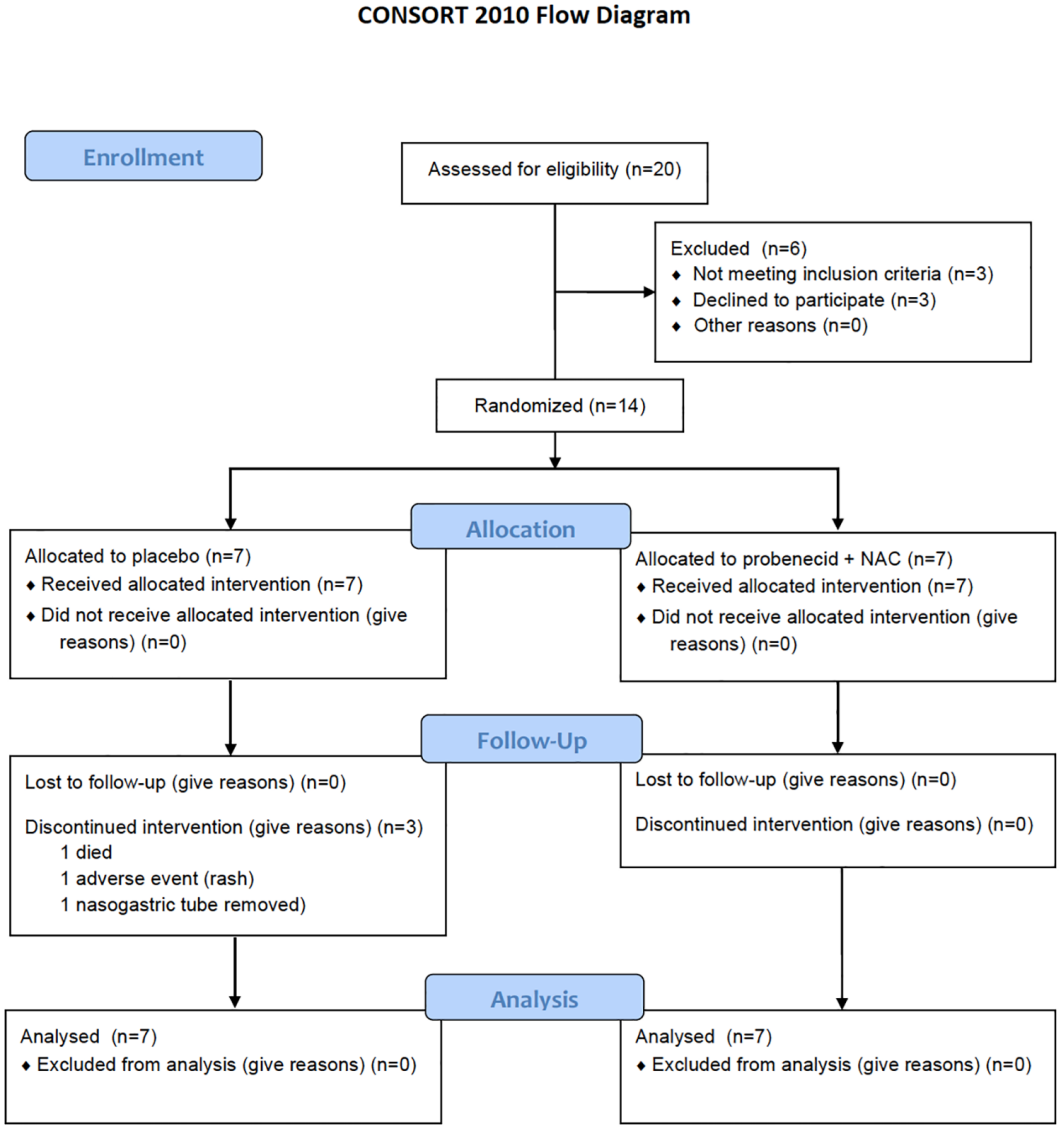
CONSORT flow diagram. *in one patient support was withdrawn due to poor neurological prognosis; one patient developed a diffuse rash after administration of study drugs and further administration was discontinued; in one patient the nasogastric tube was removed after extubation and discontinuation of mechanical ventilation, and was not replaced as per study protocol preventing further administration of study drugs.

**Fig 2:**
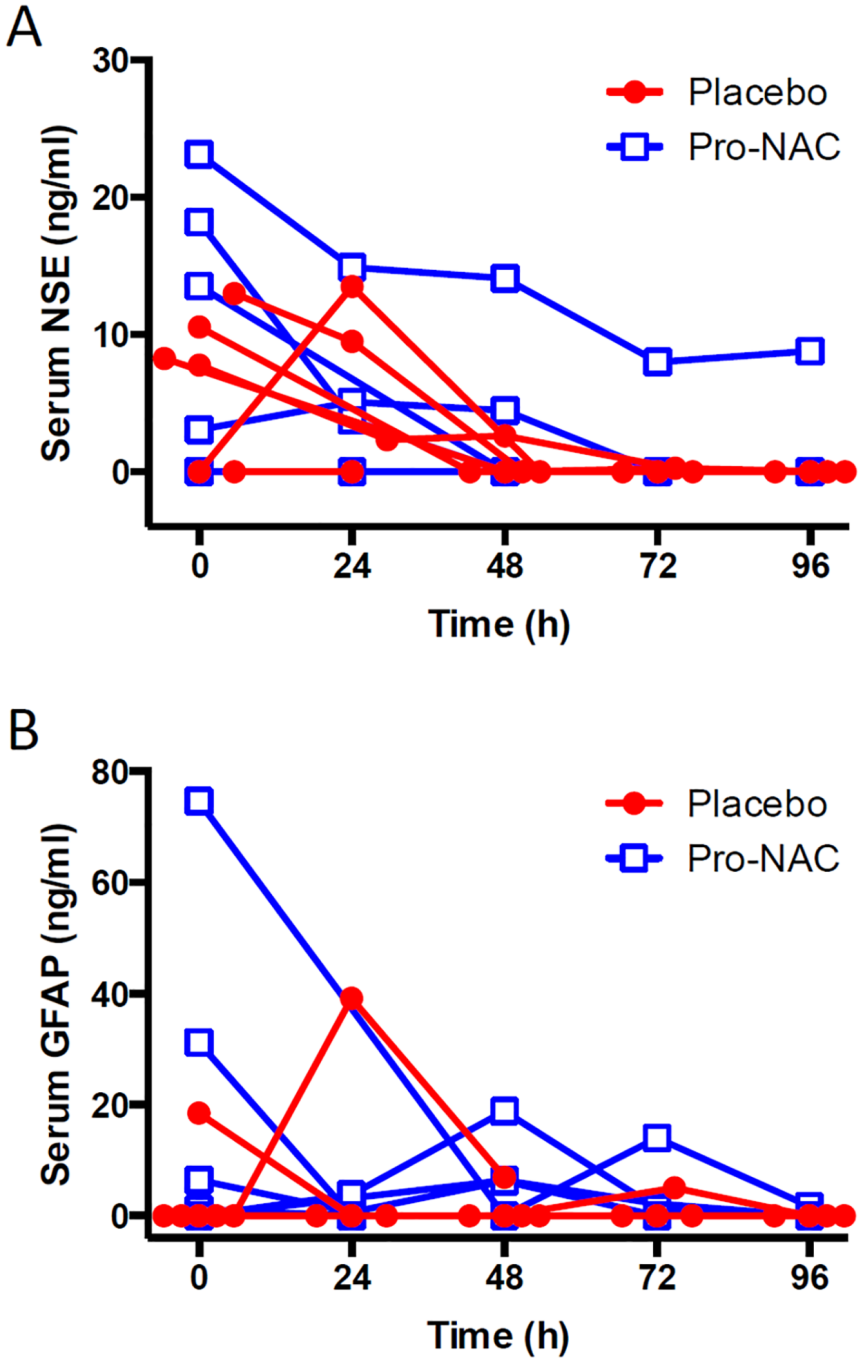
Serum brain injury biomarkers neuron specific enolase (NSE) and glial fibrillary acidic protein (GFAP) during study period. Individual NSE corrected for the degree of hemolysis (*A*) and GFAP (*B*) values for patients in the placebo (red circles) and probenecid + NAC (Pro-NAC; open blue squares) groups during the study period. There were no differences between groups for NSE (*P* = 0.441) or GFAP (*P* = 0.596). Time 0 is in reference to drug administration. Both groups started with seven patients/group. Sample attrition due to patient death, discontinuation of continuous monitoring, or insufficient quantity of serum available to perform the assay.

Patients were recruited from November 1, 2011 to August 31, 2013. Twenty patients were screened for eligibility, Seventeen patients met inclusion criteria and 14 patients were randomized after obtaining informed consent, with seven patients randomized to each group (Fig 1). Baseline characteristics of the intention-to-treat population were similar (Table 1). All seven patients receiving probenecid in combination with NAC (Pro-NAC) completed the study. However, there was attrition of patients in the placebo group related to withdrawal of life support due to neurological prognosis (n = 1), withdrawal from the study due to development of a rash occurring after administration of study drug (n = 1), and removal of the nasogastric tube prior to study completion (n = 1), resulting in four of seven patients completing the course of drug/placebo administration. Surgical interventions (Table 2) and medications (Table 3) used for the management of severe TBI were similar between groups. There were no adverse events attributable to Pro-NAC administration (Table 4). Information regarding protocol compliance is provided in Table 5. Serum levels of NSE and GFAP, interrogated using linear mixed models in order to determine if drug therapy was associated with increasing concentration or duration of these brain injury biomarkers, were not different between treatment groups (NSE *F*(1,45) = 0.60, *P* = 0.441; GFAP *F*(1,45) = 0.29, *P* = 0.596; Fig 2). Time and group interactions related to serum biomarker concentrations were also not detected (NSE *F*(4,45) = 0.08, *P* = 0.989; GFAP *F*(4,45) = 1.06, *P* = 0.387).

**Table 1:** Baseline characteristics in the intention to-treat-population.

	Placebo (n = 7)	Probenecid + NAC (n = 7)
**Age (years), mean (SD)**	9.7 (5.7)	8.6 (4.9)
**Height (cm), mean (SD)**	131.1 (32.5)	129.0 (31.3)
**Weight (kg), mean (SD)**	35.4 (20.0)	37.9 (21.3)
**Male sex**	4 (57%)	6 (86%)
**White race**	7 (100%)	6 (86%)
**Glasgow Coma Scale score**^a^, **median (IQR)**		
Composite	6 (3)	6 (2)
Eye	1 (0)	1 (0)
Verbal	1 (0)	1 (0)
Motor	4 (3)	4 (2)
**Mechanism of injury**		
Passenger in motor vehicle collision	3 (43%)	2 (29%)
Pedestrian struck by motor vehicle	0	1 (14%)
All-terrain vehicle accident	1 (14%)	0
Fall	0	1 (14%)
Other recreational activity	1 (14%)	0
Other	2 (29%)	3 (43%)

Data are n (%) unless otherwise stated.

^a^Score ranges from 1–4 for Eye, 1–5 for Verbal, 1–6 for Motor, and 3–15 for cumulative score.

**Table 2:** Surgical interventions.

	Placebo (n = 7)	Probenecid + NAC (n = 7)
**External ventricular drain placement**	7 (100%)	7 (100%)
**Evacuation of hematoma/lesion**	1 (14%)	3 (43%)
**Decompressive craniectomy**		
Unilateral	1 (14%)	3 (43%)
Bilateral	2 (29%)	0

Data are n (%).

**Table 3:** Medical interventions.

	Placebo (n = 7)	Probenecid + NAC (n = 7)
**Muscle relaxants: vecuronium**	7 (100%)	7 (100%)
**Narcotics: fentanyl**	7 (100%)	7 (100%)
**Anticonvulsants: phenytoin, fosphenytoin**	7 (100%)	7 (100%)
**Vasoactive medications: epinephrine, norepinephrine, phenylephrine**	4 (57%)	4 (57%)
**Mannitol (g)**^a^, **median (IQR)**		
Day 1	25.0 (9.5)	60.0 (50.0)
Day 2	50.0 (0.0)	0
Day 3	0	0
Day 4	0	0
Day 5	0	0
**3% hypertonic saline (ml)**^a^, **median (IQR)**		
Day 1	510.0 (1652.0)	645.0 (922.0)
Day 2	242.5 (411.0)	776.0 (699.0)
Day 3	605.0 (702.5)	663.0 (734.0)
Day 4	395.0 (515.0)	817.5 (453.0)
Day 5	360.0 (295.0)	780.0 (910.0)
**Pentobarbital (mg)**^a^, **median (IQR)**		
Day 1	1075.3 (549.4)	507.0 (614.0)
Day 2	1590.8 (1945.0)	0
Day 3	737.5 (96.2)	250.0 (0.0)
Day 4	2225.0 (0.0)	450.0 (700.0)
Day 5	3050.0 (0.0)	1027.0 (2310.0)

Data are n (%) unless otherwise stated.

^a^Cumulative daily dose for all patients in group.

**Table 4:** Adverse events and complications.

	Placebo (n = 7)	Probenecid + NAC (n = 7)
**Serious adverse event**		
Death after redirection of care and withdrawal of life support	1 (14%)	0
**CNS-related adverse event/complication**		
Refractory intracranial hypertension	1 (14%)	0
Ruptured aneurysm	0	1 (14%)
Hydrocephalus	0	1 (14%)
Vocal cord paralysis	1 (14%)	0
Diabetes insipidus	1 (14%)	0
**Other adverse events/complication**		
Acute renal failure related to vancomycin toxicity	2 (29%)	0
Pneumonia	1 (14%)	1 (14%)
Tracheitis	3 (43%)	2 (29%)
Rash	1 (14%)	0
Refractory hypotension	1 (14%)	0
Pneumothorax	1 (14%)	0
Deep venous thrombosis	1 (14%)	3 (43%)

Data are n (%).

**Table 5:** Information on protocol compliance.

	Placebo (n = 7)	Probenecid + NAC (n = 7)
**Prematurely exited the study**	3 (43%)	0
Death^a^	1 (14%)	0
Adverse event—rash^b^	1 (14%)	0
Nasogastric tube discontinued^c^	1 (14%)	0
**Drug administration error**	4 (57%)	5 (71%)
Administered at wrong time	2 (29%)	5 (71%)
Drugs not dispensed by pharmacy	0	1 (14%)
Patient in operating room during administration time	1 (14%)	0

Data are n (%).

^a^Support withdrawn due to poor neurological prognosis

^b^Developed a diffuse rash after administration of study drugs and further administration was discontinued.

^c^Nasogastric tube was removed after extubation and discontinuation of mechanical ventilation, and was not replaced as per study protocol preventing further administration of study drugs.

**Fig 3:**
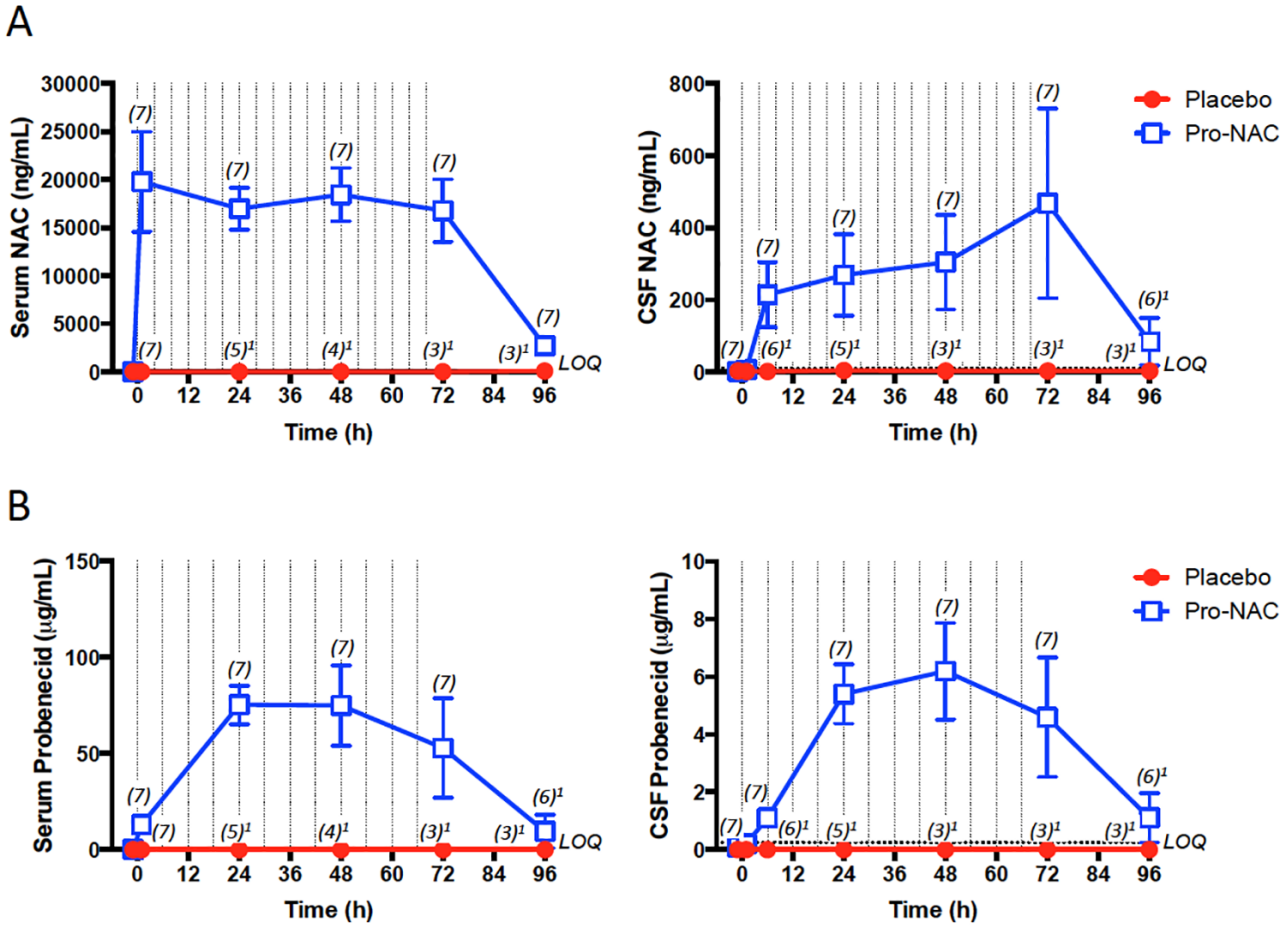
Serum and cerebrospinal fluid (CSF) N-acetylcysteine (NAC) and probenecid concentrations during study period. NAC (*A*) and probenecid (*B*) concentrations measured using ultra-high performance liquid chromatography-mass spectrometry (MS)/MS are shown for the placebo (red circles) and probenecid + NAC (Pro-NAC; open blue squares) groups. Data are mean and SEM; sample sizes noted in parentheses (n); LOQ = lower limit of quantification for the assay. Hashed lines represent drug administration times. ^1^Sample attrition due to patient death, premature withdrawal from study due to adverse event (rash), inability to continue enteric administration of study drugs after removal of nasogastric tube, and/or removal of or inability to obtain CSF from externalized ventricular drain.

Fig 3*A* shows serum and CSF NAC concentrations in the two groups. One hour after the 140 mg/kg enteral NAC loading dose, serum NAC concentrations increased to 19,789 ± 5,193 ng/mL (mean ± SEM) and appeared to reach steady state levels after 24 h (16,977 ± 2,212 ng/mL at 24 h; 18,447 ± 2,774 ng/mL at 48 h, and 16,786 ± 3,285 ng/mL at 72 h). Six hours after the NAC loading dose, CSF NAC concentrations increased to 215 ± 90 ng/mL and continued to rise during the treatment period to 269 ± 113 at 24 h, 304 ± 131 at 48 h, and 468 ± 263 ng/mL at 72 h. CSF concentrations representing 1.63%, 1.51%, and 2.52% of simultaneous serum concentrations were achieved at 24, 48, and 72 h, respectively. Predictably, NAC concentrations were below the lower limits of quantification in serum and CSF from the placebo treated group, with the exception of one patient who had a serum NAC level of 274 ng/mL (undetectable in CSF), in whom aerosolized N-acetylcysteine was being used to mobilize respiratory secretions while tracheally intubated.

Fig 3*B* shows serum and CSF probenecid concentrations in the two groups. One hour after the 25 mg/kg enteral probenecid loading dose, serum probenecid concentrations increased to 13.1 ± 3.1 μg/mL (mean ± SEM) and appeared to reach steady state after 24 h (75.4 ± 210.0 μg/mL at 24 h; 75.1 ± 20.9 μg/mL at 48 h; and 52.9 ± 25.8 μg/mL at 72 h). Six hours after the probenecid loading dose, CSF probenecid concentrations increased to 1.1 ± 0.2 μg/mL. CSF probenecid concentrations followed a similar concentration-time course as serum and were 5.4 ± 1.0, 6.2 ± 1.7, and 4.6 ± 2.1 μg/mL at 24, 48, and72 h, respectively. CSF concentrations representing 7.33%, 7.64%, and 10.16% of simultaneous serum concentrations were achieved at 24, 48, and 72 h, respectively. Predictably, probenecid levels were below the lower limits of quantification in serum and CSF from the placebo treated group.

**Fig 4:**
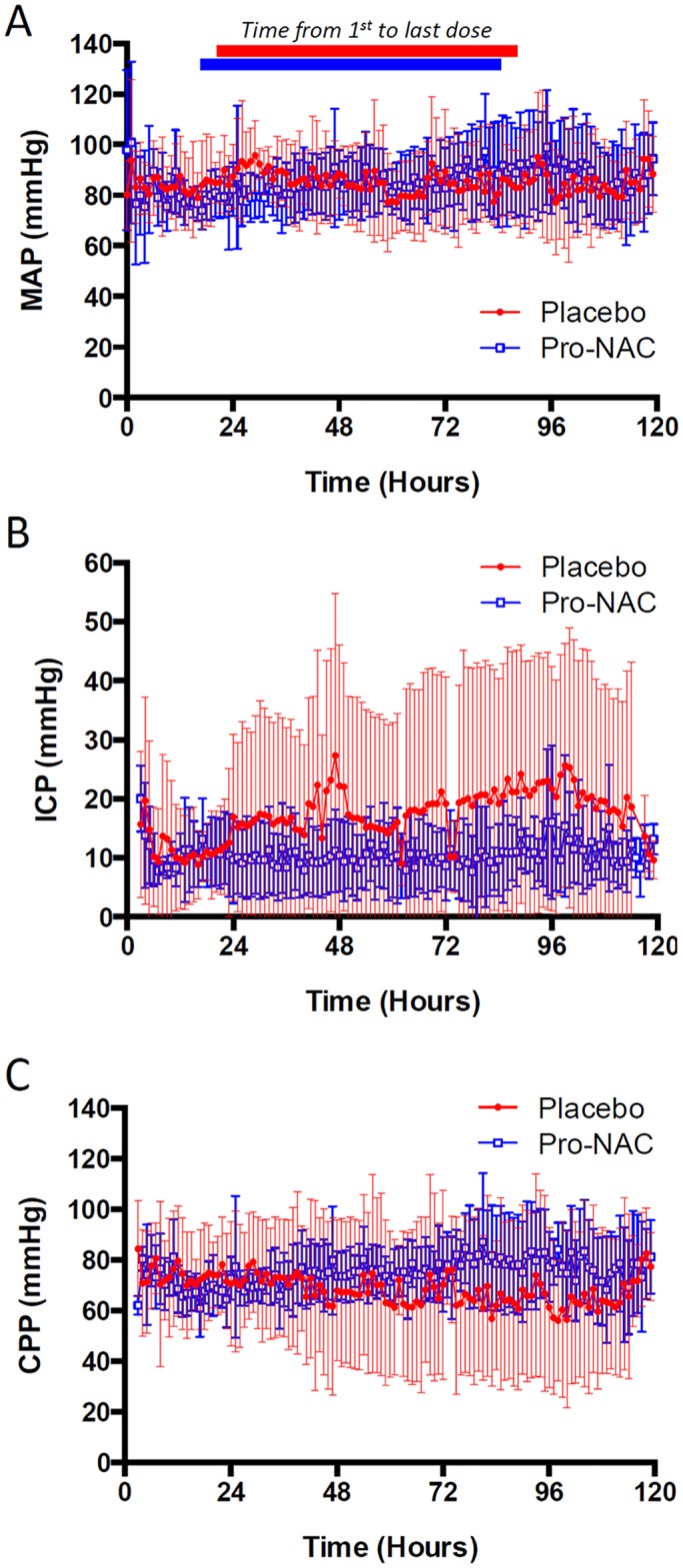
Mean arterial pressure (MAP), intracranial pressure (ICP), and cerebral perfusion pressure (CPP) during study period. MAP (*A*), ICP (*B*), and CPP (*C*) in the placebo (red circles) and probenecid + NAC (Pro-NAC; open blue squares) groups during the study period. Time 0 refers to pediatric intensive care unit admission. Median time [range] to first drug dose for the placebo group was 20.5 [14.6–26.0] h and for the Pro-NAC group was 16.5 [8.8–22.0] h. Data are mean and SD; Both groups started with seven patients/group. Sample attrition due to patient death or discontinuation of continuous monitoring.

In addition to adverse events and drug concentrations in serum and CSF, physiologic and other clinical variables were also recorded. There were no differences between groups in rectal temperature, MAP, ICP, or CPP during the study period (Fig 4). Note that ICP values in the placebo group are driven by a single patient with refractory intracranial hypertension as evidenced by 95 hourly ICP recorded values > 20 mmHg, and to a lesser extent two other patients with 14 and 21 hourly ICP recorded values > 20 mmHg. In contrast, none of the patients in the Pro-NAC group had more than 10 ICP recorded values > 20 mmHg. There were no differences between groups in GOS recorded upon hospital discharge or at 3 month follow up (Table 6). Two patients in each group were lost to follow up at 3 months. There were also no differences between groups in PICU or hospital length of stay (Table 6).

**Table 6:** Patient outcomes.

	Placebo (n = 7)	Probenecid + NAC (n = 7)	*P*
**Glasgow outcome scale score on hospital discharge**			1.000
Good recovery	1 (14%)	1 (14%)	
Moderate disability	3 (43%)	4 (57%)	
Severe disability	2 (29%)	2 (29%)	
Vegetative state	0	0	
Death	1 (14%)	0	
Unknown	0	0	
**Glasgow outcome scale score at 3 month follow up**			0.532
Good recovery	3 (43%)	1 (14%)	
Moderate disability	0	2 (29%)	
Severe disability	1 (14%)	2 (29%)	
Vegetative state	0	0	
Death	1 (14%)	0	
Unknown	2 (29%)	2 (29%)	
**PICU length of stay (days), median (IQR)**	10.0 (3.0)	13.0 (16.0)	0.485
**Hospital length of stay (days), median (IQR)**	15.0 (11.0)	21.0 (19.0)	0.533

Data are n (%) unless otherwise stated.

## Discussion

It has been estimated that the average time for translation of therapies from laboratory to patient is 17 years, a gap that is being scrutinized as being unacceptably wide [21]. Our rationale for fast-tracking the combination of probenecid and NAC for pediatric TBI was as follows: 1) each drug has been shown to be neuroprotective on its own [4, 5, 22, 23]; 2) the combination has the potential to be synergistic as probenecid and NAC preserve intracellular GSH through distinct mechanisms, i.e. preventing efflux of GSH via inhibition of ABCC1 [16] and serving as a cysteine donor for synthesis of GSH [6], respectively; 3) probenecid increases systemic and brain exposure of NAC via inhibition of OAT1 and OAT3 [14] in the kidney and brain, respectively; and 4) both probenecid and NAC are FDA approved and have favorable safety profiles, justifying repurposing for use in severe TBI given its considerable mortality and morbidity [1]. This study provides proof-of-concept that treatment with the combination of NAC and probenecid is feasible and results in readily detectable CNS concentrations of both drugs in children with severe TBI. In addition, no adverse effects or undesirable physiological derangements related to administration of the drug combination were observed in this study, although sample sizes were limited.

NAC has been, or currently is being, evaluated in clinical trials targeting neurological diseases including TBI [9–13], despite the fact that NAC is very hydrophilic (logD -5.4) [6, 7] and therefore would be predicted to have limited capacity to cross the intact BBB. In the present study, NAC achieved apparent steady state in serum rapidly (Fig 3), consistent with known terminal half-lives of NAC [24, 25]. While it was anticipated based on pharmacokinetic data in healthy adults [26] that probenecid would also approach steady state, this was not the case. Probenecid concentrations were quite variable between patients, particularly at 72 h (Fig 3). Inter-individual variability in serum probenecid concentration compared with NAC could be explained by the weight-based dosing maximum of 500 mg, longer half-life, and/or frequency of dosing. Furthermore, variable pharmacokinetics in these critically ill children would not be unexpected due to their heterogeneous injuries and multifaceted pharmacologic and non-pharmacologic interventions in the PICU, expected to impact clearance and/or enteral absorption.

For probenecid, the CSF concentration-time profile mirrors that seen in serum, but this was not the case for NAC. This apparent lag from serum to CSF observed for NAC is consistent with poor BBB permeability or transporter interactions. With co-administration of probenecid in rats, NAC levels are detectable for up to 8 h in normal brain and the area under the curve (AUC) for NAC in brain is 2.4-fold (and in serum 1.7-fold) higher versus administration of NAC alone, suggesting that NAC is a substrate for transporters inhibited by probenecid, later revealed to be OAT1 and OAT3 [14], found in both brain and kidney [15]. Accordingly, in addition to increasing GSH levels via distinct mechanisms, the combination of probenecid and NAC appear to increase blood and brain exposure of NAC via reduced renal excretion and transport-mediated brain efflux.

To our knowledge, only one other published study has reported CSF NAC concentrations in humans. Katz et al. treated 12 patients with Parkinson’s disease with oral NAC doses ranging from 7 to 70 mg/kg twice daily for two days [27]. Peak CSF NAC concentrations observed 90 min after the final dose in the 70 mg/kg treated group of adults in that study were approximately 4-fold higher than what we observed at steady state in children (Fig 2). However, in the Parkinson’s disease study [27], NAC peaks were measured and given NAC elimination is two-compartmental, levels between studies are not readily comparable. Furthermore, the regimen for NAC in the present study was based on oral dosing for treatment of acetaminophen/paracetamol overdose. Steady state trough serum NAC concentrations achieved in our study are expected to be higher than in other studies of NAC alone based on reduced renal excretion related to probenecid sensitive transporters.

Probenecid was developed as an antibiotic adjuvant during World War II to reduce OAT-mediated renal excretion of penicillin for treatment of wound infections in soldiers [28]. In human kidney slices the inhibitory constant (Ki) values for probenecid tested with a specific OAT3 probe (benzylpenicillin) was 3.6 μg/mL [29]. Probenecid concentrations observed in our pediatric patients superseded this in both serum and CSF (Fig 3). Thus, probenecid levels in serum and CSF appear sufficient to achieve inhibition of OATs impacting pharmacokinetics of NAC. One important caveat related to the use of probenecid is that it would inhibit transport of a spectrum of endogenous organic anion or xenobiotic substrates of OAT1 and OAT3. The endogenous organic anions could represent both desirable and undesirable substances in the CSF and thus may impact the brain’s extracellular biochemical milieu. A comprehensive metabolomics profile using remaining CSF from this study is underway to begin to address this.

Given that this study was appropriately powered to serve as a phase I pharmacokinetic study, the sample size was sufficient to define the primary goal of drug levels, but it was not designed to be able to inform on acute or chronic outcomes, and is thus only exploratory for those parameters. Another limitation to the present study includes measurement of total NAC and not reduced NAC. Thus, our measurements include oxidized NAC and all NAC conjugates. Reduced NAC levels may be more pharmacologically active. However, the pharmacokinetic portion of our study was specifically designed to determine overall exposure following dosing, and total NAC measurement is ideal in this regard. Finally, we did not include a NAC-only treatment arm in this pilot study, and given the number of studies using NAC for treatment of neurological disease [9–13], follow up trials for severe TBI should include a NAC alone arm in either a balanced or response-adaptive design.

To our knowledge, this study represents: 1) the first reported use of probenecid as an adjuvant for medications unrelated to their antimicrobial activity; 2) the first CNS pharmacokinetic study of NAC and/or probenecid in children; and 3) the first CNS pharmacokinetic trial in pediatric TBI of any kind. Treatment with the combination of NAC and probenecid resulted in readily detectable CNS levels of both drugs in children with severe TBI. Given that adverse effects including undesirable physiological derangements and alterations in contemporary brain injury biomarkers related to administration of the drug combination were not observed, progression to a phase II/III trial appears warranted.

## Supporting information

S1 Table Pre-defined adverse events. (DOCX)

S1 Fig Pro-NAC CONSORT flow diagram. (DOCX)

S1 Checklist Pro-NAC CONSORT checklist. (DOC)

S1 Protocol Pro-NAC clinical study protocol. (PDF)
