# Supplementary material for: Phase I randomized clinical trial of N-acetylcysteine in combination with an adjuvant probenecid for treatment of severe traumatic brain injury in children
Source: PLoS One. 2017 Jul 7;12(7):e0180280. doi: 10.1371/journal.pone.0180280 (PMC5501440; doi:10.1371/journal.pone.0180280)
Supplement: S1 Table — (DOCX) [file pone.0180280.s001.docx]

**S1 Table. Pre-defined adverse events.**

| Acute renal failure  Anaphylaxis  Acute respiratory distress syndrome  Intracranial infection/abscess  Arrhythmia, atrial  Arrhythmia, ventricular  Bradycardia  Cardiac arrest  Catheter positive culture  Cerebrospinal fluid leak  Decubitis  Deep vein thrombosis  Diabetes Insipidus  Emesis  Extraaxial hematoma  Gastrointestinal bleed  Gastritis  Hematuria  Hemorrhage, other  Hemoperitonium  Hemothorax  Hepatitis  Hydrocephalus  Hypotension  Hypoxemia  Infection, other  Intraparenchymal hemorrhage  Intraventricular hemorrhage  Meningitis/ventriculitis  Multiorgan dysfunction syndrome  Myocardial ischemia  Pancreatitis  Pericarditis  Peritonitis  Pneumothorax  Pulmonary edema  Pulmonary embolism  Respiratory arrest  Seizures  Sepsis  Syndrome of inappropriate antidiuretic hormone  Transtentorial herniation  Withdrawal of Life Support  Other SAE causing re-hospitalization  Other SAE |
| --- |
