# Supplementary material for: Phase I randomized clinical trial of N-acetylcysteine in combination with an adjuvant probenecid for treatment of severe traumatic brain injury in children
Source: PLoS One. 2017 Jul 7;12(7):e0180280. doi: 10.1371/journal.pone.0180280 (PMC5501440; doi:10.1371/journal.pone.0180280)
Supplement: S1 Fig — (DOCX) [file pone.0180280.s002.docx]

**CONSORT 2010 Flow Diagram**

## Allocation

Analysed (n=7)
♦ Excluded from analysis (give reasons) (n=0)

## Analysis

Lost to follow-up (give reasons) (n=0)

Discontinued intervention (give reasons) (n=3)

1 died

1 adverse event (rash)

1 nasogastric tube removed)

Allocated to placebo (n=7)

♦ Received allocated intervention (n=7)

♦ Did not receive allocated intervention (give reasons) (n=0)

Analysed (n=7)
♦ Excluded from analysis (give reasons) (n=0)

## Follow-Up

Lost to follow-up (give reasons) (n=0)

Discontinued intervention (give reasons) (n=0)

## Enrollment

Allocated to probenecid + NAC (n=7)

♦ Received allocated intervention (n=7)

♦ Did not receive allocated intervention (give reasons) (n=0)

Randomized (n=14)

Excluded (n=6)

♦  Not meeting inclusion criteria (n=3)

♦  Declined to participate (n=3)

♦  Other reasons (n=0)

Assessed for eligibility (n=20)
