# Supplementary material for: Phase I randomized clinical trial of N-acetylcysteine in combination with an adjuvant probenecid for treatment of severe traumatic brain injury in children
Source: PLoS One. 2017 Jul 7;12(7):e0180280. doi: 10.1371/journal.pone.0180280 (PMC5501440; doi:10.1371/journal.pone.0180280)
Supplement: S1 Protocol — (PDF) [file pone.0180280.s004.pdf]

Pro-NAC I Trial

Phase I Safety/Pharmacokinetics Trial for the Treatment of Traumatic Brain Injury in Children with the Combination of Probenecid and N-acetylcysteine

Clinical PI: Michael J. Bell, MD
PI: Robert S. B. Clark, MD
Research coordinator: Rachelle L. Bell, RN

Table of Contents **Page**

I. Study overview……………………………………………………………………………... 2

II. Patient enrollment, drug treatments, and sample collection…………………….............. 2

III. PICU management …………………………………………………………………………. 3

IV. Study outcome measurements……………………………………………………………. 5

V. Data safety and monitoring………………………………………………………………… 6

VI. References …………………………………………………………………………………. 6

Appendices

APPENDIX A: Study procedures and responsibilities………………………………………… 8
APPENDIX B: Study Screening Form (SCRN)………………………………………................ 10
APPENDIX C: Serious Adverse Events (SAE)………………………………………………… 12
APPENDIX D: Adverse Events (ADVEV)……………………………………………………….. 15
APPENDIX E: Acute Care: ICU Stay – Medications (MEDIC)……………………………….. 16
APPENDIX F: Cranial Surgery Form (SURG)…………………………………………………. 19
APPENDIX G: Protocol Deviations Form (PRDEV)…………………………………………… 20
APPENDIX H: Clinical Protocol Deviation (CPD)……………………………………………… 21
APPENDIX I: Acute Care ICU Stay – Physiology (PHYSO)………………………………….. 23
APPENDIX J: Glasgow Outcome Scale (GOS)………………………………………………… 24
APPENDIX K: Hospital Discharge Form (DISCH)…………………………………………….. 25
APPENDIX L: Acute Care ICU Stay – Labs (LABS)…………………………………………… 26
APPENDIX M: Neurological Exam (NEURO)………………………………………………….. 30

I. Study overview

PHASE I SAFETY/PHARMACOKINETICS TRIAL FOR THE TREATMENT OF TRAUMATIC BRAIN INJURY IN CHILDREN WITH THE COMBINATION OF PROBENECID AND N-ACETYLCYSTEINE

Overview
We plan to enroll 20 children (n = 10/group) age 2-18 y admitted to the Pediatric Intensive Care Unit (PICU) at the Children's Hospital of Pittsburgh after severe TBI (GCS < 8) with indwelling extraventricular drains (EVD), arterial lines, and continuous EEG placed as part of standard management. Patients will be randomized to receive **probenecid (initial: 25 mg/kg/dose; maintenance: 10 mg/kg/dose 4 x per day for 11 doses) and NAC (initial: 140 mg/kg/dose; maintenance: 70 mg/kg/dose 6 x per day for 17 doses)** or **placebo** via nasogastric (NG) or orogastric (OG) tube for 3 days.

Inclusion Criteria
1. Admission to the PICU for treatment of severe TBI
2. GCS < 8 prior to or after ICU admission
3. Age 2 to 18 years at the time of ICU admission
4. Presence of indwelling EVD, arterial catheters, and NG/OG tube with expected duration of > 2 days

Exclusion Criteria
1. Brain dead on admission to ICU
2. Pregnancy
3. Contraindications to enteral medications
4. *Contraindications to probenecid*: a) status epilepticus; b) blood dyscrasias; c) under 2 years-of-age; d) co-administration of salicylates; e) renal dysfunction or urate kidney stones; f) hypersensitivity to probenecid
5. *Contraindications to N-acetylcysteine*: hypersensitivity to N-acetylcysteine
6. Family unwilling to consent

**No inclusions or exclusions will be based on gender or ethnicity*

Measurements
1. Daily CSF and serum levels of probenecid, NAC, GSH, phenytoin and fentanyl (possible MRP substrates), and total AOR for 5 days
2. Daily urine uric acid (probenecid should reduce concentrations) and NAC for 5 days
3. Physiological parameters including temperature, mean arterial pressure (MAP), arterial blood gases, intracranial pressure (ICP), and EEG, will be collected as part of standard practice
4. All adverse events, whether attributable to study drugs or not
5. GCS and Glasgow Outcome Score (GOS) upon hospital discharge
6. Genotyping for common MRP1 polymorphisms

II. Patient enrollment, drug treatments, and sample collection

Planned enrollment: Twenty children age 2-18 y admitted to the PICU at CHP after severe TBI (GCS < 8). Inclusion criteria: (1) Admission to the PICU for treatment of severe TBI; (2) GCS < 8 prior to or after ICU admission; (3) age 2 to 18 years at the time of PICU admission; (4) presence of indwelling EVD, arterial catheter, and NG/OG tube with expected duration of > 2 days. Exclusion Criteria: (1) Brain dead on admission to ICU; (2) pregnancy; (3) contraindications to enteral medications; (4) contraindications to probenecid: a) status epilepticus; b) blood dyscrasias; c) under 2 years-of-age; d) co-administration of salicylates; e) renal dysfunction (creatinine > 1 under 12 y.o., > 1.5 over 12 y.o.) or uric acid kidney stones; f) hypersensitivity to probenecid; (5) contraindications to N-acetylcysteine: hypersensitivity to N-acetylcysteine; (6) family unwilling to consent. **No inclusions or exclusions will be based on gender or ethnicity.*

Consent and randomization: For eligible patients, the legal guardian(s) will be approached by the principal investigator (Dr. Clark) or clinical principal investigator (Dr. Bell), who will provide IRB-approved informed consent. Time from injury will not be a determining factor in terms of eligibility; however, those patients where it

is predicted that the EVD and other intensive care monitoring will not be in place for > 2 days from enrollment will be excluded since this will result in missing data points for the pharmacokinetic studies. If the patient's legal guardian(s) agree to consent to participate, the patient will be randomized to study drugs or placebo using randomly sorted sealed envelopes containing group assignments. *Participation in the study will not influence clinical management of the patient.*

Study drugs and administration:
Probenecid: Initial dose 25 mg/kg, maintenance dose 10 mg/kg/dose 4 x per day for 11 doses (maximum single dose 500 mg), crushed and suspended in 10 ml normal saline, delivered via NG or OG tube for 3 days

N-acetylcysteine: Initial 140 mg/kg/dose, maintenance 70 mg/kg/dose 6 x per day for 17 doses, 200 mg/ml solution delivered via NG or OG tube for 3 days.

Placebo: Equal volumes and dosing regimens of lactose powder (for opacity) suspended in Ora-Plus® and normal saline delivered via NG or OG tube for 3 days.

Study drugs or placebo will be prepared by a dedicated Research Pharmacist, and delivered to the patient's bedside labeled as "Study Drug A", "Study Drug B", "Study Drug Y", or "Study Drug Z", and will be delivered by the PICU nurse caring for the patient. *Patients will not be charged for study drugs or pharmacy support.*

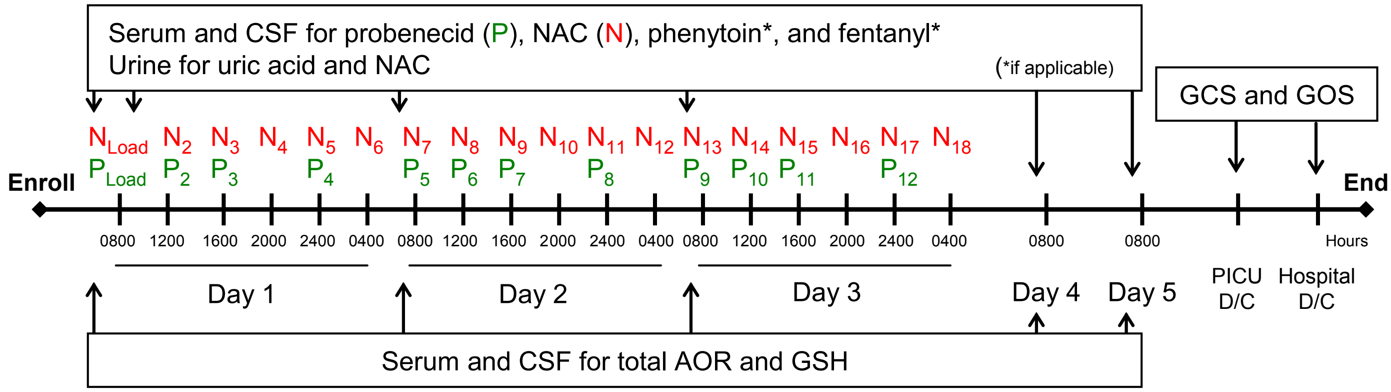

III. PICU management

All patients will be treated based on specific protocols adopted from the Guidelines for Acute Medical Management following Severe TBI in Infants, Children, and Adolescents, a document endorsed by the American Association for Surgery of Trauma, Child Neurology Society, International Society for Pediatric Neurosurgery, International Trauma Anesthesia and Critical Care Society, Society of Critical Care Medicine, and the World Federation of Pediatric Intensive Care Societies, and published in *Pediatric Critical Care Medicine* and the *Journal of Trauma* in 2012 [1].

For TBI patients with a GCS < 8 as part of standard of care in the PICU, an ICP monitor is routinely placed and is typically in the form of an EVD, an arterial line is placed for continuous monitoring of MAP and blood gas sampling, a central venous catheter is placed to monitor central venous pressure (CVP), a 6-channel continuous EEG is placed to detect seizures, and an OG or NG is placed to decompress the stomach and administer medications. The only standard exception to these contemporary neurointensive care practices are for patients who are clinically brain dead upon PICU admission. The severe TBI protocol for physiologic support includes: head of bed at 0-30°; isotonic crystalloid (without dextrose), colloid or blood products as indicated for fluid expansion to treat hypotension and/or shock, continuous monitoring of systemic arterial O_2 saturation, MAP, and pulse. Enteral or parenteral nutrition is initiated 48 h after injury. The primary therapeutic goals in the PICU are to maintain: MAP within 1 standard deviation for age, systemic arterial O_2 saturation > 94%, ICP < 18 mmHg (< 6 y), ≤ 20 mmHg (≥ 6 y), cerebral perfusion pressure (CPP) > MAP (for age) - 20 mmHg, and core body temperature at 36.5- 37.5°C (except those randomized to hypothermia). To determine the presence of surgical intracranial mass lesions after the initial CT scan, patients undergo a repeat head CT

scan immediately after any sudden, severe rise in ICP as per standard protocol. *Duration of invasive monitoring will not be influenced by this study, i.e. maintenance of EVD or vascular catheters solely for the purpose of obtaining CSF or serum samples for this study will be prohibited and strictly enforced.*

<u>ICP monitoring and management of intracranial hypertension:</u> **An indwelling EVD for ICP monitoring and drainage of CSF is required for patients to be eligible for the study, since CSF total AOR and GSH are primary outcome variables, and drug levels are essential for this pharmacokinetic study.** The following tiered protocol attempts to maintain age appropriate CPP and ICP, and will be initiated for ICP > 18 or 20 mmHg for > 5 min in children aged < 6 y or ≥ 6 y, respectively. ICP measurements will be monitored continuously and will be recorded on an hourly basis. The tiered protocol will be followed in a stepwise manner with failure of one therapy dictating escalation to the next therapy in the algorithm:

1. Head in a neutral position at 0-30° elevation
2. Continuous infusion of a narcotic for patient comfort with or without neuromuscular blockade (vecuronium)
3. Intermittent and/or continuous ventricular drainage of CSF
4. Osmotherapy: mannitol, initially at 0.25 g/kg q 4- 6 h as needed for ICP elevations, with the option to increase to 0.5-1 g/kg until serum osmolality ≥ 320 mOsm **and/or** hypertonic saline (3%) via continuous infusion initiated at 0.1-0.5 ml/kg/h and titrated to effective ICP control or a maximum serum sodium of 155 mEq/L
5. Barbiturates, initially 5 mg/kg/dose q 4- 6 h, with escalation to continuous infusion targeting 80-90% burst suppression on continuous EEG monitoring **and/or** surgical intervention, either decompressive craniectomy, lumbar drain, and/or temporal lobectomy for failure of medical management

The mean, median, and percent time ICP > 18 or 20 mmHg for the different age groups will be calculated during the treatment period for each group. **A greater percentage of time above ICP threshold values (i.e. intracranial hypertension) is associated with poor outcome, and will serve as a secondary outcome variable, along with systemic hypotension or hypertension, for safety monitoring.**

<u>Mechanical ventilation:</u> All patients with severe TBI are endotracheally intubated and supported with mechanical ventilation. $PaCO_2$ will be routinely maintained at 35-38 mmHg during the study period by adjusting minute ventilation. Hyperventilation in the management of TBI is generally avoided [2]. To maintain O_2 saturation > 94%, FiO_2 and/or positive end expiratory pressure will be adjusted. For periods of acute intracranial hypertension, hyperventilation to $PaCO_2$ < 34 mmHg may be used transiently until other measures can be instituted.

<u>Standard laboratory studies</u>: Patients admitted to the PICU for management of severe TBI are monitored with daily (or more frequently as needed) complete blood counts with differential and platelets (CBC), prothrombin and partial thromboplastin times (PT/PTT), serum basic metabolic profiles (BMP includes Na^+, K^+, Cl^-, creatinine, blood urea nitrogen (BUN), glucose), Ca^{2+}, Mg^{2+}, PO_4, and daily CSF cell counts. Evaluation of liver function tests and pancreatic enzymes are also done on admission and as needed. All patients on fosphenytoin have serum phenytoin levels checked daily. Chest radiographs are done daily in part to screen for ventilator associated pneumonia. Additional surveillance for nosocomial infection in the form of blood, CSF, tracheal, and urine cultures are done based on clinical suspicion.

<u>PICU and hospital discharge</u>: *Patient readiness for PICU or hospital discharge will not be influenced by participation in this study.* Discharge from the PICU will be based on resolution of intracranial hypertension, i.e. no longer receiving therapies directed at ICP including CSF drainage, as well as stability of cardiovascular and pulmonary status, and nursing considerations. Typically patients are transferred from the PICU to the general neurosurgical unit, but occasionally they are transferred directly to a Rehabilitation facility. The decision to transfer to a Rehabilitation facility or discharge home is made by the Neurosurgical and Trauma Services. The length of stay in the PICU and acute care facility will be documented.

Study-specific sample collection: All patient samples will be collected by the bedside PICU nurse using aseptic technique. CSF samples (10 ml if available) will be collected from the EVD reservoir, centrifuged at 5000 x*g* for 10 min to remove cellular material. CSF from the reservoir would otherwise be discarded. Serum samples (5 ml) will be collected from the arterial or central venous catheters, and centrifuged to separate serum from cellular material (the pellet may be used for DNA extraction). Urine (10 ml) will be collected from the urinary bladder catheter reservoir. CSF, serum and urine samples will be obtained from the bedside by the research nurse (Danielle Brown), aliquoted into microcentrifuge tubes for use in separate assays, and frozen at -80°C until batch analysis.

IV. Study outcome measurements

Outcome measurements specifically related to the study are shown in **Table 1**. **The primary outcome variables will be serum and CSF concentrations of NAC and probenecid and adverse events.** Secondary outcome variables include: daily CSF and serum levels of GSH and AOR for 5 days; TBI-related physiological parameters including ICP, CPP, MAP, heart rate, and temperature; GCS on admission and on PICU and hospital discharge; GOS upon hospital discharge; and all adverse events, whether attributable to study drugs or not. Surveillance for adverse events including infection, electrolyte disturbances, blood dyscrasias, renal dysfunction, hepatic dysfunction, and elevation of pancreatic enzymes will occur as part of standard of care as described in the above section.

Table. Summary of study outcome measures

Variable	Admission	Day 1	Day 2	Day 3	Day 4	Day 5	PICU discharge	Hospital discharge
CSF and serum NAC and probenecid		X[1]	X	X	X	X		
Adverse events (including seizures[2])	----X----	---X---	---X---	---X---	---X---	---X---	-------X-------	--------X--------
CSF and serum AOR and GSH		X	X	X	X	X		
ICP, CPP, and MAP	----X----	---X---	---X---	---X---	---X---	---X---		
Glasgow Coma Scale score	X						X	X
Glasgow Outcome Score							X	X

[1]CSF and serum samples drawn prior to study drug administration
[2]Clinical or on EEG

Probenecid: Concentrations of probenecid will be measured in CSF and serum. Emanuelsson et al. have reported probenecid levels of 192 ± 23 µg/ml in serum and 9.3 ± 2.9 µg/ml in CSF 14-20 h after a 4.5 g oral dose in healthy human volunteers [3]. Thus, at least 5% appears to cross the BBB after oral administration.

NAC: Concentrations of NAC will be measured in CSF and serum using UPLC-MS/MS. Celma et al. [4] have reported the capacity to measure NAC concentrations from 50-1000 ng/ml in serum from human volunteers.

AOR: Total AOR will be measured in CSF and serum as described [5].

GSH: GSH will be measured in CSF and serum as described [5].

TBI-specific physiologic variables: Continuous ICP and other physiologic data during the study period will be captured using the Bedmaster EX system (Excel Medical Electronics, Inc., Jupiter, FL) linked to the hospital's computer network. Briefly, this system links all free-standing bedside devices (including ICP monitors, brain oxygen monitors, near-infrared spectroscopy, etc.) with the bedside monitor for each patient. Patient-derived physiological data every will be acquired every 5 sec and stored into a password-protected database as the source document for all neurocritical care studies. Within this database, average hourly values for physiological variables can be calculated. The presence of any episodic abnormality of interest (i.e., ICP > 20 mmHg for more than 5 min) can be retrieved for all study patients. This system downloads all data into Microsoft Excel spreadsheets that will serve as the template for incorporation into Data Collection Forms for the Pediatric Neurotrauma Center database at the School of Public Health.

GCS: Initial GCS will be abstracted from Emergency Medical Service (EMS) and Emergency Department records. GCS upon PICU and hospital discharge will be assigned by the Neurosurgery Service, who will be unaware of treatment group. These data will be collected and reported, but not statistically analyzed.

GOS: The GOS is a standard outcome measure in adults with TBI that is widely accepted, despite low sensitivity. GOS upon hospital discharge will be assigned by the Neurosurgery Service, who will be unaware of treatment group. Using the categories: 1 = death, 2 = vegetative, 3 = moderate disability, 4 = mild disability, and 5 = normal. These data will be collected and reported, but not statistically analyzed.

Data handling: Dr. Clark (PI) and Dr. Bell (Clinical PI) will oversee all aspects of the clinical study, and be responsible for the confidential collection, integration, and dissemination of data. Once all samples and clinical data are collected, the data will be de-identified to ensure patient confidentiality. Rachelle Bell (Study Coordinator) will collect and archive all relevant demographic data, prior to de-identification. Statistical analysis will be overseen by Dr. Stephen Wisniewski.

Handling of missing data points and attrition: Missing data points e.g. due to catheter malfunction, inability to collect samples because of patient instability, and attrition e.g. due to early removal of catheter or death of the patient, are not uncommon in this cohort of critically ill children. Based on the average survival to hospital discharge (88.5%) and average length of PICU stay (6.5 days), we expect attrition to be ~10%. We do not intend to enroll patients where it is felt the duration of EVD placement due to severity of illness is to be < 3 days.

V. Data safety and monitoring

An independent, multidisciplinary DSMB has been formed to assure subject safety and human subject protection (see Appended materials for confirmation of participation). The DSMB will review the Informed Consent Form and IRB prior to submission, and will meet annually. In addition, every adverse event detected by the physicians, nurses, and/or ancillary staff primarily caring for a patient within the study will be reported to the PI (Dr. Clark) or Clinical-PI (Dr. Bell), who will assign the event to at least two members of the DSMB. These members will determine whether the event may be attributable to one of the study drugs, or the study itself. Reviewing DSMB members will call a meeting of the DSMB as a whole, along with the investigators, if an event is felt to be possibly or likely related to the study. The DSMB has the authority to suspend or terminate the study at any time. If this occurs, the PI will notify the IRB, Institution (CHP), FDA, and NIH within 24 h.

DSMB members
Barbara A. Gaines, MD. Director of Trauma and Injury Prevention, Children's Hospital of Pittsburgh of UPMC, Associate Professor of Surgery, University of Pittsburgh School of Medicine

Carol G. Schmitt, PharmD. Clinical Pharmacy Specialist, Children's Hospital of Pittsburgh

Ann E. Thompson, MD. Director, Pediatric Intensive Care Unit, Children's Hospital of Pittsburgh of UPMC, Professor and Vice-Chair of Critical Care Medicine, University of Pittsburgh School of Medicine

Elizabeth Tyler-Kabara, MD, PhD. Assistant Professor of Neurological Surgery and Bioengineering, University of Pittsburgh School of Medicine

VI. References

1. Kochanek PM, Carney N, Adelson PD, Ashwal S, Bell MJ, Bratton S, et al. Guidelines for the acute medical management of severe traumatic brain injury in infants, children, and adolescents--second edition. Pediatr Crit Care Med. 2012;13 Suppl 1:S1-82.
2. Muizelaar JP, Marmarou A, Ward JD, Kontos HA, Choi SC, Becker DP, et al. Adverse effects of prolonged hyperventilation in patients with severe head injury: A randomized clinical trial. J Neurosurg. 1991;75:731-9.

3. Emanuelsson BM, Widerlov E, Walleus H, Paalzow LK. Determinations of 5-hydroxyindoleacetic acid and homovanillic acid in human CSF with monitoring of probenecid levels in CSF and plasma. Psychopharmacology (Berl). 1987;92(2):144-9.
4. Celma C, Allue JA, Prunonosa J, Peraire C, Obach R. Determination of N-acetylcysteine in human plasma by liquid chromatography coupled to tandem mass spectrometry. J Chromatogr A. 2000;870(1-2):13-22.
5. Bayir H, Kagan VE, Tyurina YY, Tyurin V, Ruppel RA, Adelson PD, et al. Assessment of antioxidant reserves and oxidative stress in cerebrospinal fluid after severe traumatic brain injury in infants and children. Pediatr Res. 2002;51(5):571-8.

APPENDIX A: ProNAC Study procedures and responsibilities

Subject Screening – Clinical PI, PI, and Study Coordinator

- Respond to Level 1 Trauma page or to CCM Fellow Phone Call regarding any TBI admitted to the PICU
- Inclusion Criteria: diagnosis of severe TBI, GCS ≤ 8, presence of EVD, arterial/venous lines and gastric tube, age 2 y – 18 y
- Exclusion Criteria: brain dead at admission, pregnant, contraindication to enteral medications, contraindications to probenecid (status epilepticus, blood dyscrasias, salicylates, renal dysfunction or urate kidney stones, hypersensitivity to probenecid, meropenem), hypersensitivity to NAC, lack of informed consent from both parents
- Child must be enrolled and drug administered within 24 h after TBI
- We will try to enroll children as soon as is feasible
- Time from TBI till first drug is administered will be tracked for further analysis
- Patient will be assigned a study number and orders will be prepared for pharmacy (paper orders at first, transitioning to electronic orders)

Randomization and Drug Administration – Research Pharmacist Responsibilities

- Research pharmacist will be responsible for preparation of placebos and drugs or will be responsible for providing necessary information for clinical pharmacists
- Orders for enrolled patients will be sent from PICU to pharmacy along with study ID number
- Pharmacists will open randomization envelopes for the study
- 20 Randomization Envelopes will be provided by the Data Management and Coordinating Center (DMAC)
- Pharmacists will chose one envelope, administer either both drugs or both placebos and maintain records regarding which drug was administered
- Total of 11 doses of probenecid and 17 doses of NAC (or placebo) will be administered based on the drug schedule and will be prepared based on recipe provided by research pharmacist (probenecid - initial: 25 mg/kg/dose; maintenance: 10mg/kg/dose 4 x per day for 11 doses; and NAC -initial: 140mg/kg/dose; maintenance: 70mg/kg/dose 6 x per day for 17 doses)
- Drugs will be delivered to patient bedside by usual mechanisms
- Bedside nurses will administer via NG tube, flush with 5 cc sterile water and clamp NG tube for 1 h
- Bedside nurse will record exact time that dose was delivered

Biological Sample Collection – Clinical PI and Study Coordinator

- Serum and CSF will be collected at baseline (before any drug/placebo administration), 1 h after first dose of drug, 24 h after baseline, 48 h after baseline, 72 h after baseline and 96 h after baseline (note: all times are after baseline, not after TBI itself).
- Five (5) cc of blood will be placed in a red-top tube and allowed to stand for 10 minutes. Then, sample will be centrifuged at 10 min @ 3000g until serum is separated. Samples will be aliquoted into 2 eppendorf tubes. Five (5) cc of CSF will be collected from the buretrol. If enough CSF is not available, EVD will be lowered to 3 cm above midbrain for sufficient time to acquire the CSF sample. CSF will be similarly centrifuged and aliquoted as the serum. Serum and CSF will be frozen at -20°C until transported to the laboratories in Oakland.
- Baseline and 1 h urine samples will be obtained directly from foley bag and immediately frozen at -20°C. 24 h urine collections will be begun at the bedside with the urine placed on ice. 24 h, 48 h, 72 h and 96 h urine specimens will be obtained from these urine collections and an aliquot of 5 cc will be frozen. Urine will be transported to appropriate laboratory in Oakland.
- Serum/CSF/Urine will be transported to the School of Pharmacy at Falk Hall. Serum/CSF will also be transported to 2nd Avenue Laboratory of Dr. Bayir.

Data Collection and Analysis – Clinical PI and Study Coordinator

- Study Coordinator will complete all data collection forms and be responsible for entering data into database.

- Clinical PI and Study Coordinator will evaluate for adverse events/serious adverse events every day of the study up until hospital discharge.
- Discharge and 3 month GOSePeds will be determined by reviewing medical records.
- Reports will be prepared for DSMB review on a quarterly basis.
- Data from Bayir/Poloyac/Empey labs will be generated and put into the database for analysis.
- After 20 subjects enrolled, analysis will be completed for all study parameters.

APPENDIX B: Study Screening Form (SCRN)

Inclusion Criteria

1. ☐ Yes ☐ No Admission to the PICU for treatment of severe traumatic brain injury

2. ☐ Yes ☐ No Glasgow Coma Scale (GCS) < 8 (prior to or after admit to ICU) **Glasgow Coma Score: ___**

3. ☐ Yes ☐ No Presence of indwelling EVD, arterial/venous catheters, and NG/OG tube with expected duration of > 2 days

4. ☐ Yes ☐ No Age 2 – <18 years old (< 216 m) at time of ICU admission

If any of the above items are "No", the patient is not eligible for enrollment. Skip to #11 and check "No".

Exclusion Criteria ("complete ONLY if all Inclusion Criteria are "Yes")

5. ☐ Yes ☐ No Brain dead on admission to the ICU

6. ☐ Yes ☐ No Pregnancy (must be documented by a urine pregnancy test for females > 10 years of age)
(Check "NO" if patient is 10 years or younger or 'MALE')

7. ☐ Yes ☐ No Contraindications to enteral medications

8. ☐ Yes ☐ No Contraindications to probenecid: a) status epilepticus; b) blood dyscrasias; c) under 2 years of age; d) co-administration of salicylates; e) renal dysfunction or urate kidney stones; f) hypersensitivity to probenecid

9. ☐ Yes ☐ No Contraindications to N-acetylcysteine: a) hypersensitivity to N-acetylcysteine

10. ☐ Yes ☐ No Family unwilling to consent or unable to obtain consent from <u>both</u> parents (unless one parent is deceased, unknown, incompetent, or not reasonably available; or when only one parent
has
legal responsibility for the child)

No inclusions or exclusions will be based on gender or ethnicity

---

↓

11. ☐ Yes ☐ No **Is the patient eligible for randomization based on the inclusion and exclusion criteria?**
(Patient is eligible for randomization if all Inclusion Criteria are "Yes" and all Exclusion Criteria are "No".)

↓

Patient is INELIGIBLE for enrollment.
STOP HERE (do not complete the rest of the form).

Consent Information

12. ☐ Yes ☐ No Has informed consent been obtained ?

↓

Please note reason why consent was not obtained:

- ☐ Parent / Guardian is unavailable
- ☐ Inability to obtain signed consent (i.e., non-English speaking)
- ☐ Refused consent. Specify reason:
  - ☐ Does not want child to be a research participant
  - ☐ Does not want child to receive a randomized treatment
  - ☐ Other, specify reason: ______________________________________

    ____
- ☐ Other, specify: ____

Patient is INELIGIBLE.
STOP HERE (do not complete the rest of the form).
Enter the form into MATRIX to document ineligibility.

Enter date and time consent for study enrollment was obtained:

Date of Consent for *study enrollment*: __ __ / __ __ / __ __ __ __ (mm/dd/yyyy)

Time of Consent (use 24- hour clock): __ __ : __ __

13. ☐ Yes ☐ No **Is the patient eligible for randomization based on the consent information?**

(Patient is eligible for randomization if informed consent is obtained)

Patient is INELIGIBLE for enrollment.
STOP HERE (do not complete the rest of the form).
Enter the form into MATRIX to document ineligibility.

Randomization Information

The patient is eligible for study enrollment and may be randomized by using the blind envelope system.
Upon selection of an envelope please open and record the patient's study ID and randomized treatment assignment

PATIENT STUDY ID: _______________________________

PATIENT ASSIGNED TO: ☐ PLACEBO ☐ DRUG THERAPY

SAE Date of Onset: __ __ / __ __ / __ __ __ __

Time of Onset: __ __ : __ __

SAE Status: ☐ Continuing
☐ Controlled
☐ Resolved
If Controlled or Resolved: Resolution Date: __ __ / __ __ / __ __ __ __

Serious Adverse Event:

☐ Acute Renal Failure
☐ Anaphylaxis
☐ ARDS (Acute Respiratory Distress Syndrome)
☐ Arrhythmia, atrial
☐ Arrhythmia, ventricular
☐ Bradycardia
☐ Cardiac arrest
☐ Catheter positive culture
☐ CSF Leak
☐ Decubitis
☐ Deep vein thrombosis
☐ Diabetes Insipidus
☐ Emesis
☐ Extraaxial Hemotoma
☐ GI Bleed
☐ Gastritis
☐ Hematuria
☐ Hemorrhage, other
☐ Hemoperitonium
☐ Hemothorax
☐ Hepatitis
☐ Hydrocephalus
☐ Hypoxemia
☐ Infection, other
☐ Intracranial infection/abcess
☐ Intraparenchymal hemorrhage
☐ Intraventricular hemorrhage
☐ Meningitis/ventriculitis
☐ MODS
☐ Myocardial ischemia
☐ Pancreatitis
☐ Pericarditis
☐ Peritonitis
☐ Pneumothorax
☐ Pulmonary edema
☐ Pulmonary embolism
☐ Respiratory arrest
☐ Seizures
☐ Sepsis
☐ Syndrome of inappropriate ADH
☐ Transtentorial herniation
☐ Withdrawal of Life Support
☐ Other SAE causing re-hospitalization _______________
☐ Other SAE:___________________________________
☐ Hypotension

Relationship to treatment:

☐ Unrelated (clearly not related to the research)
☐ Unlikely (doubtfully related to the research)
☐ Possible (may be related to the research)
☐ Probable (likely related to the research)
☐ Definitely (clearly related to the research)

Expected? ☐ Yes ☐ No

SAE Outcome: ☐ Death (**If Death, please complete the Death of Subject Form)**

☐ Life-threatening event
☐ Hospitalization *(prolonged or new)*
☐ Disability
☐ Congenital anomaly
☐ Other, specify:_____________________________________

To ensure that the blinded status of study personnel is maintained, please be sure NOT to include any information related to the specific treatment assignment received by the patient (placebo or drug) when completing the items on this page.

Describe event:
____

____
_

____
_

____
_

Medications/Therapies used to treat SAE:
____

____
_

____
_

Record any related tests or laboratory data, including dates:____

____
_

____
_

____
_

____
_

List any concomitant medications:____

-
____
_

____
_

____
_
Any relevant history including pre-existing medical conditions?
____

____
_

-
____
_

Comments:
____

____
_

____
_

____ Date signed: __ __ / __ __ / __ __ __ __
Principal Investigator's Signature mm dd yyyy

APPENDIX D: Adverse Events (ADVEV)

Adverse Event	Start Date	Stop Date	Severity	Related to Study Treatment?	Treated?	AE Continuing?
			☐ Mild<br>☐ Moderate	☐ Unrelated ☐ Unlikely<br>☐ Possible ☐ Probable ☐ Definite	☐ Yes<br>☐ No	☐ Resolved<br>☐ Persistent
			☐ Mild<br>☐ Moderate	☐ Unrelated ☐ Unlikely<br>☐ Possible ☐ Probable ☐ Definite	☐ Yes<br>☐ No	☐ Resolved<br>☐ Persistent
			☐ Mild<br>☐ Moderate	☐ Unrelated ☐ Unlikely<br>☐ Possible ☐ Probable ☐ Definite	☐ Yes<br>☐ No	☐ Resolved<br>☐ Persistent
			☐ Mild<br>☐ Moderate	☐ Unrelated ☐ Unlikely<br>☐ Possible ☐ Probable ☐ Definite	☐ Yes<br>☐ No	☐ Resolved<br>☐ Persistent
			☐ Mild<br>☐ Moderate	☐ Unrelated ☐ Unlikely<br>☐ Possible ☐ Probable ☐ Definite	☐ Yes<br>☐ No	☐ Resolved<br>☐ Persistent
			☐ Mild<br>☐ Moderate	☐ Unrelated ☐ Unlikely<br>☐ Possible ☐ Probable ☐ Definite	☐ Yes<br>☐ No	☐ Resolved<br>☐ Persistent
			☐ Mild<br>☐ Moderate	☐ Unrelated ☐ Unlikely<br>☐ Possible ☐ Probable ☐ Definite	☐ Yes<br>☐ No	☐ Resolved<br>☐ Persistent
			☐ Mild<br>☐ Moderate	☐ Unrelated ☐ Unlikely<br>☐ Possible ☐ Probable ☐ Definite	☐ Yes<br>☐ No	☐ Resolved<br>☐ Persistent
			☐ Mild<br>☐ Moderate	☐ Unrelated ☐ Unlikely<br>☐ Possible ☐ Probable ☐ Definite	☐ Yes<br>☐ No	☐ Resolved<br>☐ Persistent
			☐ Mild<br>☐ Moderate	☐ Unrelated ☐ Unlikely<br>☐ Possible ☐ Probable ☐ Definite	☐ Yes<br>☐ No	☐ Resolved<br>☐ Persistent

APPENDIX E: Acute Care: ICU Stay – Medications (MEDIC)

Date __ __ /__ __ /__ __ __ __ **PICU Day # __ __**

Paralytics Yes No

If yes, check all that apply

Vecuronium
Pancuronium (Pancuronium bromide)
Rocuronium (Zemuron)
Succinylcholine
Paralytic, Other Please Specify ________________________

Narcotics/Sedation Yes No

If yes, check all that apply

Fentanyl (Duragesic, Sublimaze)
Morphine (Astramorph, Duramorph)
Propofol (Diprivan)
Ativan (Lorazepam)
Diazepam (Valium)
Demerol (Meperidine)
Hydromorphone (Dilaudid)
Dexmetomidate
Methadone (Dolophine)
Midazolam (Versed)
Oxycodone (OxyIr, Percolone, Roxicodone)
Sedative, Other Please Specify ________________________

Anticonvulsant Yes No

If yes, check all that apply

Phenytoin (Dilantin)
Fosphenytoin (Cerebyx)
Keppra (Levetiracetam)
Phenobarbital (Luminal Sodium, Nembutal Sodium)
Oxcarbazepine (Trileptal)
Primidone (Mysoline)
Topiramate (Topamax)
Carbamazepine (Tegretol)
Valproic acid (Depakene)
Anticonvulsant, Other Please Specify ________________________

Vasoactive Yes No

If yes, check all that apply

Dobutamine (Dobutrex)
Dopamine (Intropin)
Epinephrine (Adrenalin)
Isoproterenol (Isuprel)
Labetelol (Trandate)
Nitroglycerin (Nitrogard, Nitro-Bid, Tridil)
Nitroprusside (Nitropress)
Norepinephrine (Levophed)
Phenylephrine (Neo-Synephrine)
Vasoactive, Other Please Specify _______________________

Antibiotic/Antifungal/Antiviral Yes No

If yes, check all that apply

Amikacin (Amikin)
Amphotericin B (Ambisome, Amphocin, Amphotec, Fungizone)
Ampicillin (Omnipen)
Cefazolin (Ancef, Kefzol, Zolicef)
Ceftazidime (Ceptaz, Fortaz, Tanicef)
Ceftriaxone (Rocephin)
Ciprofloxacin (Cipro)
Clindamycin (Cleocin)
Fluconazole (Diflucan)
Ganciclovir (Vitrasert)
Gentamicin (Garamycin)
Ketoconazole (Nizoral)
Meropenem (Merrem)
Oxacillin (Prostaphilin, Bactocil)
Pipercillin (Pipracil)
Pipercillin/Tazo (Zozyn)
Ticarcillin
Tobramycin (Tobrex, Nebcin)
Vancomycin (Vancocin)
Antibiotic, other Please Specify ___________________________
Antifungal, other Please Specify ___________________________
Antiviral, other Please Specify ___________________________

Other Yes No

If yes, check all that apply

Atropine
Clonidine (Catapres)

Lidocaine (Lidamantle, Xylocaine)
Potassium chloride (Kaon, Rum-K)
Potassium phosphate (Neutra-Phos-K)
Propranolol (Inderal)
Insulin, regular
Sodium bicarbonate
Methylprednisolone
Steroid, other
Thiopental (Pentothal)
Vassopressin (Pitressin)
Other Please Specify ___________________________

Mannitol (Osmitrol) Yes No
If yes, total dose in grams in 24 hours ____________

Hypertonic Saline Yes No
If yes, total dose in cc in 24 hours ____________

Pentobarbital (Nembutal) Yes No
If yes, total dose in mg in 24 hours ____________

Diuretic Yes No

APPENDIX F: Cranial Surgery Form (SURG)

Complete one form per OR visit.

Surgery Date: __ __ /__ __ /__ __ __ __

Surgery Time __ __ : __ __ (24-hour clock)

Check all that apply:

☐ Yes ☐ No **Intracranial monitor placement?**

Check all that apply:

☐ Yes ☐ No **EVD placement**

☐ Yes ☐ No **Intraparenchymal catheter placement**

☐ Yes ☐ No **Placement of other monitor**

Specify other monitor: ______________________________________

☐ Yes ☐ No **Evacuation of lesion**

Check all that apply:

☐ Yes ☐ No **Epidural**

☐ Yes ☐ No **Subdural**

☐ Yes ☐ No **Intraparenchymal**

☐ Yes ☐ No **Decompressive craniectomy**

Specify: ☐ Yes ☐ No **Bilateral**

☐ Yes ☐ No **Unilateral**

☐ Yes ☐ No **Other, specify:** ____

EARLY STUDY EXIT

1. Did the patient prematurely exit the study? ☐ **Yes** ☐ **No**

↓

☐ **Yes** ☐ **No** Was the drug protocol aborted or altered?

If Yes, When: __ __ / __ __ / __ __ __ __

Specify reason: **mm dd yyyy**

☐ Brain Death
☐ Life Support Withdrawn
☐ Other, Specify

☐ **Yes** ☐ **No** Withdrawal of informed consent by parent/guardian

If Yes, Date informed consent was withdrawn: __ __ / __ __ / __ __ __ __

Check all that apply: **mm dd yyyy**

☐ **Yes** ☐ **No** Patient/family is moving
☐ **Yes** ☐ **No** Research is too burdensome
☐ **Yes** ☐ **No** No reason given
☐ **Yes** ☐ **No** Other, Specify:

PATIENT ENROLLMENT

2. **Were there protocol deviations related to enrollment of the patient?** ☐ **Yes** ☐ **No**

↓

Check all that apply:

Yes	No	
☐	☐	Randomization of an ineligible patient
☐	☐	Failure to obtain informed consent
☐	☐	Participant was entered into another intervention study
☐	☐	Wrong treatment assignment was administered
☐	☐	Other, specify:

APPENDIX H: Clinical Protocol Deviation (CPD)

Date of call: __ __ / __ __ / __ __ __ __

Time of call: __ __ : __ __ (24-hr clock)

Who made the call: ☐ Site PI ☐ Site Coordinator ☐ Other, specify:
____

Request type: ☐ Post-hoc deviation notification ☐ Deviation request

↓

Date of Clinical Deviation: __ __ / __ __ / __ __ __ __

Time of Clinical Deviation: __ __ : __ __ (24-

Describe event:
____

____

____

Deviation (what was done or is being requested?):

____

____

____

Reason for deviation:
____

____

____

Resolution: Deviation approved? ☐ Yes ☐ No

Study PI Comments:

____

____

_

____

_

____ Date signed: __ __ / __ __ / __ __ __ __

Coordinating Center Signature mm dd yyyy

APPENDIX I: Acute Care ICU Stay – Physiology (PHYSO)

ICU Day # 1 ***NOTE THE CPP WILL BE AUTOMATICALLY CALCULATED IN MATRIX***

Hour #	Date (mm/dd /yyyy)	Time (24-hr clock)	HR (Beats/min)	mmHg					ºC	
				MAP	ICP Ventricular	ICP Interparenchymal	CPP	CVP	Temp Rectal	Temp Brain
0										
1										
2										
3										
4										
5										
6										
7										
8										
9										
10										
11										
12										
13										
14										
15										
16										
17										
18										
19										
20										
21										
22										
23										

APPENDIX J: Glasgow Outcome Scale (GOS)

NOTE: Only problems that have **developed or become markedly worse** since the head injury should be considered when completing the GOS. That is, the child's premorbid status must be weighed when answering all questions.

Information obtained: ☐ From records

Please check the category below that most closely represents the patient's current level of functioning based upon the most reliable data gathered during the interview measures. This rating is the patient's GOS score.

☐ **1) Good Recovery –** This indicates the capacity to resume normal occupational and social activities, although there may be minor physical or mental deficits or complaints. Full recovery without signs or symptoms is included here. However, for various reasons (other than mental or physical deficit), the patient may not have resumed all his previous activities, and in particular, may not be working.

☐ **2) Moderate Disability –** These patients may be summarized as "independent but disabled," but is perhaps the least easily described category of survivor. These patients have independence of activities of daily living; for instance, can travel by public transport and look after themselves at home. However, some previous activities, either at work or in their social life, are now no longer possible by reason of either physical or mental deficit. Despite evident posttraumatic signs, resumption of activities at a lower level is often possible or posttraumatic signs are present which, however, allow resumption of most former activities, whether full-time or part-time. Some patients in this category are unable to return to certain kinds of work, if this happens to involve a high level of performance in the area of their major deficit.

☐ **3) Severe Disability –** Due to many posttraumatic complaints and/or deficits, resumption of former life and work are not possible. Communication is possible, minimally by emotional response. This indicates that a patient is conscious but needs the assistance of another person for some activities every day. Total or almost total dependency with regard to activities of daily living life or partial independence in activities of daily life exists. This may range from continuous total dependency to the need for assistance with only one activity, such as dressing. More often dependency is due to the combination of physical and mental disability because when physical disability is severe after head injury, there is almost always considerable mental deficit. But a few patients who have little or no physical deficit are unable to organize their day-to-day lives effectively, and must be classified as severely disabled. The worst of these requires the care and protection which only a mental hospital can provide, while others cope at home with the support of attentive relatives, but could not be left overnight because they would be unable to plan their meals or to deal with callers, or any domestic crisis which might arise. The severely disabled are described by the phrase "conscious but dependent."

☐ **4) Vegetative State –** Non-sentient not obeying commands, no verbal contact, no meaningful response; they may have sleep-wake rhythm. These are patients who show no evidence of meaningful responsiveness. Vegetative patients breathe spontaneously, have periods of spontaneous eye opening when they may follow moving objects with their eyes, show reflex response in their limbs (to postural and painful stimuli), and they may swallow food placed in their mouths. This non-sentient state must be distinguished from other conditions of wakeful, reduced responsiveness, such as locked-in syndrome, akinetic mutism, and total global aphasia. Although the vegetative state indicates a lack of function in the cerebral cortex, this may be structurally intact.

☐ **5) Death.**

APPENDIX K: Hospital Discharge Form (DISCH)

Date of hospital discharge (or death): __ __ /__ __ /__ __ __ __

Date of ICU discharge: __ __ /__ __ /__ __ __ __

Discharge Destination:

☐ Home

☐ Rehabilitation facility

☐ Skilled nursing facility

☐ Death → **Complete a "Death of Subject" form**

☐ Unknown

APPENDIX L: Acute Care ICU Stay – Labs (LABS)

ICU Day # 1

Hour #	Date (mm/dd/yyyy)	Time (24-hr clock)	PH	PO2 mm Hg	PCO2 mmHg	Base Excess mmol/L	End Tidal CO2 mmHg	PBTO2 mmHg	SO2 %	Platelets # in 1,000s/ml	HGB g/dl	HCT %	WBC # in 1,000s/ml
0													
1													
2													
3													
4													
5													
6													
7													
8													
9													
10													
11													
12													
13													
14													
15													
16													
17													
18													
19													
20													
21													
22													
23													

ICU Day # 1 (continued)

Hour #	Date (mm/dd /yyyy)	Time (24-hr clock)	NA mmol /L	K mm ol/L	D-stick Glucose (mg/dl)	Serum Glucose (mg/dl)	Serum OSM Osm/kg	PT seco nds	PTT seco nds	IN R	Pheny toin Levels	Cortiso l	Barbitur ate Level
0													
1													
2													
3													
4													
5													
6													
7													
8													
9													
10													
11													
12													
13													
14													
15													
16													
17													
18													
19													
20													
21													
22													
23													

ICU Day # 1 (continued)

Hour #	Date (mm/dd /yyyy)	Time (24-hr clock)	Serum Proben ecid	Seru m NAC	Seru m GSH	Serum phenyt oin	Serum fentan yl	Serum total AOR	Serum MRP substrate s	Urine Uric Acid Levels	Urine NAC levels	CSF Data
0												
1												
2												
3												

4												
5												
6												
7												
8												
9												
10												
11												
12												
13												
14												
15												
16												
17												
18												
19												
20												
21												
22												
23												

Hour #	Date (mm/dd/yyyy)	Time (24-hr clock)	Cholesterol	LDL	VLDL	Triglycerides	Total Protein	Albumin	Pre-albumin	Procalcitonin
0										
1										
2										
3										
4										
5										
6										
7										
8										
9										
10										
11										
12										
13										
14										

15										
16										
17										
18										
19										
20										
21										
22										
23										

APPENDIX M: Neurological Exam (NEURO)

	Admission Exam	Day 1	Day 2	Day 3	Day 4
Test Date	__ __/__ __/__ __ __ __	__ __/__ __/__ __ __ __	__ __/__ __/__ __ __ __	__ __/__ __/__ __ __ __	__ __/__ __/__ __ __ __
**Status (check all that apply)**	☐ Sedated<br>☐ Intubated	☐ Paralyzed<br>☐ Sedated<br>☐ Intubated	☐ Paralyzed<br>☐ Sedated<br>☐ Intubated	☐ Paralyzed<br>☐ Sedated<br>☐ Intubated	☐ Paralyzed<br>☐ Sedated<br>☐ Intubated
	**Note: Patient cannot be paralyzed for Admission Exam**	**Note: GCS data is not expected if the patient is Paralyzed**			
**GCS: Eye**<br>**Verbal**<br>**Motor**<br>**Total**	__<br>__<br>__<br>__ __	__<br>__<br>__<br>__ __	__<br>__<br>__<br>__ __	__<br>__<br>__<br>__ __	__<br>__<br>__<br>__ __
**Pupil Size: Right**<br>**Left**	__ mm<br>__ mm	__ mm<br>__ mm	__ mm<br>__ mm	_ mm<br>__ mm	__ mm<br>__ mm
**Pupil Reaction: Right**<br>**Left**	☐ Normal<br>☐ Sluggish<br>☐ Fixed<br>☐ Normal<br>☐ Sluggish<br>☐ Fixed	☐ Normal<br>☐ Sluggish<br>☐ Fixed<br>☐ Normal<br>☐ Sluggish<br>☐ Fixed	☐ Normal<br>☐ Sluggish<br>☐ Fixed<br>☐ Normal<br>☐ Sluggish<br>☐ Fixed	☐ Normal<br>☐ Sluggish<br>☐ Fixed<br>☐ Normal<br>☐ Sluggish<br>☐ Fixed	☐ Normal<br>☐ Sluggish<br>☐ Fixed<br>☐ Normal<br>☐ Sluggish<br>☐ Fixed
**Pupil Shape: Right**<br>**Left**	☐ Round<br>☐ Oval<br>☐ Round<br>☐ Oval	☐ Round<br>☐ Oval<br>☐ Round<br>☐ Oval	☐ Round<br>☐ Oval<br>☐ Round<br>☐ Oval	☐ Round<br>☐ Oval<br>☐ Round<br>☐ Oval	☐ Round<br>☐ Oval<br>☐ Round<br>☐ Oval
		**Note: Gaze, Corneal and Cough/Gag/Swallow data is not expected if the patient is Paralyzed**			
**Gaze**	☐ Normal<br>☐ Abnormal<br>☐ Not Tested	☐ Normal<br>☐ Abnormal<br>☐ Not Tested	☐ Normal<br>☐ Abnormal<br>☐ Not Tested	☐ Normal<br>☐ Abnormal<br>☐ Not Tested	☐ Normal<br>☐ Abnormal<br>☐ Not Tested
**Corneal**	☐ Present<br>☐ Absent<br>☐ Not Tested	☐ Present<br>☐ Absent<br>☐ Not Tested	☐ Present<br>☐ Absent<br>☐ Not Tested	☐ Present<br>☐ Absent<br>☐ Not Tested	☐ Present<br>☐ Absent<br>☐ Not Tested
**Cough/Gag/ Swallow**	☐ Normal<br>☐ Abnormal<br>☐ Not Tested	☐ Normal<br>☐ Abnormal<br>☐ Not Tested	☐ Normal<br>☐ Abnormal<br>☐ Not Tested	☐ Normal<br>☐ Abnormal<br>☐ Not Tested	☐ Normal<br>☐ Abnormal<br>☐ Not Tested
